# A Novel Model of Urinary Tract Differentiation, Tissue Regeneration, and Disease: Reprogramming Human Prostate and Bladder Cells into Induced Pluripotent Stem Cells

**DOI:** 10.1016/j.eururo.2013.03.054

**Published:** 2013-11

**Authors:** Mohammad Moad, Deepali Pal, Anastasia C. Hepburn, Stuart C. Williamson, Laura Wilson, Majlinda Lako, Lyle Armstrong, Simon W. Hayward, Omar E. Franco, Justin M. Cates, Sarah E. Fordham, Stefan Przyborski, Jane Carr-Wilkinson, Craig N. Robson, Rakesh Heer

**Affiliations:** a Northern Institute for Cancer Research, Newcastle University, UK; b Institute of Genetic Medicine, Newcastle University, UK; c Department of Urological Surgery, Vanderbilt University Medical Centre, TN, USA; d School of Biological and Biomedical Science, Durham University, UK

**Keywords:** Prostate, Bladder, Differentiation, Pluripotent, Stem cells, Tissue engineering, Ureter, Urothelium, Androgen receptor, POU5F1 (formerly OCT4), SOX2, KLF4, MYC (formerly cMYC), NANOG

## Abstract

**Background:**

Primary culture and animal and cell-line models of prostate and bladder development have limitations in describing human biology, and novel strategies that describe the full spectrum of differentiation from foetal through to ageing tissue are required. Recent advances in biology demonstrate that direct reprogramming of somatic cells into pluripotent embryonic stem cell (ESC)-like cells is possible. These cells, termed *induced pluripotent stem cells* (iPSCs), could theoretically generate adult prostate and bladder tissue, providing an alternative strategy to study differentiation.

**Objective:**

To generate human iPSCs derived from normal, ageing, human prostate (Pro-iPSC), and urinary tract (UT-iPSC) tissue and to assess their capacity for lineage-directed differentiation.

**Design, setting, and participants:**

Prostate and urinary tract stroma were transduced with POU class 5 homeobox 1 (POU5F1; formerly OCT4), SRY (sex determining region Y)-box 2 (SOX2), Kruppel-like factor 4 (gut) (KLF4), and v-myc myelocytomatosis viral oncogene homolog (avian) (MYC, formerly C-MYC) genes to generate iPSCs.

**Outcome measurements and statistical analysis:**

The potential for differentiation into prostate and bladder lineages was compared with classical skin-derived iPSCs. The student *t* test was used.

**Results and limitations:**

Successful reprogramming of prostate tissue into Pro-iPSCs and bladder and ureter into UT-iPSCs was demonstrated by characteristic ESC morphology, marker expression, and functional pluripotency in generating all three germ-layer lineages. In contrast to conventional skin-derived iPSCs, Pro-iPSCs showed a vastly increased ability to generate prostate epithelial-specific differentiation, as characterised by androgen receptor and prostate-specific antigen induction. Similarly, UT-iPSCs were shown to be more efficient than skin-derived iPSCs in undergoing bladder differentiation as demonstrated by expression of urothelial-specific markers: uroplakins, claudins, and cytokeratin; and stromal smooth muscle markers: α-smooth-muscle actin, calponin, and desmin. These disparities are likely to represent epigenetic differences between individual iPSC lines and highlight the importance of organ-specific iPSCs for tissue-specific studies.

**Conclusions:**

IPSCs provide an exciting new model to characterise mechanisms regulating prostate and bladder differentiation and to develop novel approaches to disease modelling. Regeneration of bladder cells also provides an exceptional opportunity for translational tissue engineering.

## Take Home Message

Induced pluripotent stem cells derived from human prostate and urinary tract cells can differentiate back into prostate and bladder cells. These cells provide a convenient ready-to-access model that offers enormous potential in clinical regenerative medicine and disease modelling.

## 1 Introduction

Mechanisms involved in prostate and bladder development and differentiation have been implicated in disease [1–3]. Established animal models of prostate and bladder development are well characterised; however, despite providing key insights for differentiation, there are limitations in translating these findings to the human setting. Similarly, there are constraints on studying cells lines that forcibly maintain expression of immortalising genes. Approaches to human primary cell culture can overcome some of these issues, although this can be challenging due to the limited life-span of cultures, biologic variability between patients, limited material despite cell expansion, and changes in the phenotype associated with in vitro culture adaptation [4–6]. An alternative strategy is the use of embryonic stem cells (ESCs) that can generate foetal and adult prostate and bladder tissue [7,8], but this approach is limited by ethics associated with the source of material. However, a recent landmark discovery by Yamanaka and colleagues demonstrated that somatic cells can be reset to an embryonic-like state, termed *induced pluripotent stem cells* (iPSCs), by the expression of defined factors [9,10]. Such cells offer an unparalleled opportunity for regenerative therapies, disease modelling, and drug screening [11]. However, iPSCs appear to retain epigenetic imprinting associated with their tissue type of origin. This phenomenon results in restricted terminal differentiation into other cell types [12–14].

In this study, we generated, for the first time, iPSCs derived from human prostate (Pro-iPSCs) and urinary tract cells (bladder and ureter) (UT-iPSCs). Furthermore, our data showed that Pro-iPSCs and UT-iPSCs are more efficient in differentiating into respective prostate and bladder lineages relative to established skin fibroblast-derived iPSCs, confirming the importance of the organ of origin on the differentiation potential of the reprogrammed cell.

## 2 Materials and methods

### 2.1 Cell culture

All surgical specimens were collected according to local ethical and regulatory guidelines and included written, informed patient consent (Freeman Hospital, Newcastle Upon Tyne, UK). Patient details from which successful iPSC lines were established are summarised in Table 1. Histologic examination confirmed the absence of dysplasia or malignancy. Prostate primary culture was undertaken according to previously optimised protocols to separate purified epithelial and stromal cells [15,16]. Cell cultures of urothelium and associated urinary tract stroma were established using a protocol described by Southgate et al. [17]. Detailed protocols for cell culture are provided in Supplement 1. The homogeneity of the stromal cells that were subsequently transduced were confirmed by real-time reverse transcription-polymerase chain reaction (RT-PCR) using a panel of cell lineage markers (CD24 epithelial, CD45 haematopoietic, von Willebrand factor endothelial, CD146 endothelial, α-smooth-muscle actin [SMA] stromal smooth muscle, and Thy-1 cell surface antigen [CD90] stromal cells).

### 2.2 Lentivirus transduction

Pure cultures of 5 × 10^4^ prostate, bladder, and ureter stromal cells were transduced using a polycistronic lentiviral vector (POU class 5 homeobox 1 [POU5F1, formerly OCT4], SRY [sex determining region Y]-box 2 [SOX2], Kruppel-like factor 4 (gut) [KLF4], and v-myc myelocytomatosis viral oncogene homolog [avian] [MYC, formerly C-MYC]; Allele Biotech, San Diego, CA, USA) at a multiplicity of infection of 10 in the presence of polybrene (10 μg/ml) and transduction medium (RPMI1640 medium with HEPES modification; Sigma-Aldrich Co, St. Louis, MO, USA) containing 10% foetal calf serum (Sigma-Aldrich Co, St. Louis, MO, USA), l-glutamine (2 mM), and 1% penicillin and streptomycin (Invitrogen Corp, Carlsbad, CA, USA). On day 2, the transduction medium (including lentiviral vectors) was replaced with standard stroma culture medium. On day 6, cells were seeded onto gelatine-coated plates with a feeder layer of irradiated CF-1 mouse embryonic fibroblasts (MEFs) (MTI-GlobalStem, Rockville, MD, USA) in human ESC medium (Knockout Dulbecco's modified Eagle's medium, 1 mM l-glutamine, 100 mM nonessential amino acids, 20% serum replacement, and 8 ng/ml fibroblast growth factor [FGF] 2 [Invitrogen Corp, Carlsbad, CA, USA]). Additional details on optimisation of these protocols are available in Supplement 1. After an additional week, cells were cultured in MEF-conditioned ESC medium. ESC-like colonies were manually selected based on morphology between 4 and 6 wk. The medium was changed every 48 h. A similar protocol was applied to epithelial cells but was unsuccessful in iPSCs generation; it is described in Supplement 1.

### 2.3 Characterisations by polymerase chain reaction, DNA fingerprinting, karyotyping, immunofluorescence, and alkaline phosphatase staining

RNA isolation and real time RT-PCR was normalised to glyceraldehyde 3-phosphate dehydrogenase according to protocols described in Supplement 1. DNA fingerprinting was based on microsatellite markers for short tandem repeats and karyotyping was determined by Giemsa banding. Details about this and about alkaline phosphatase activity detection and immunofluorescence are described in Supplement 1.

### 2.4 Assays of pluripotency

In vitro and in vivo differentiation assays, and embryoid body and teratoma formation were undertaken using established protocols and are detailed in Supplement 1.

### 2.5 Lineage-specific differentiation of human induced pluripotent stem cells in vitro

MEFs were removed for differentiation studies. For the induction of prostate differentiation, Pro-iPSCs were cultured with primary prostate stroma-conditioned medium. For bladder differentiation, UT-iPSCs were cultured with conditioned medium for 14 d using a previously established protocol [18]. Details of the conditioned medium from either cultured human urothelial cells or stroma cells are described in Supplement 1. Comparison of differentiation potential was drawn against conventional human-skin iPSCs, which is a pre-established cell line [19].

### 2.6 Statistical analysis

All results are expressed as mean plus or minus the standard error, and statistical differences assessed using the student *t* test with *p* values ≤0.05 considered significant.

## 3 Results

### 3.1 Generation of induced pluripotent stem cells from normal ageing prostate and human urinary tract cells

**Fig. 1:**
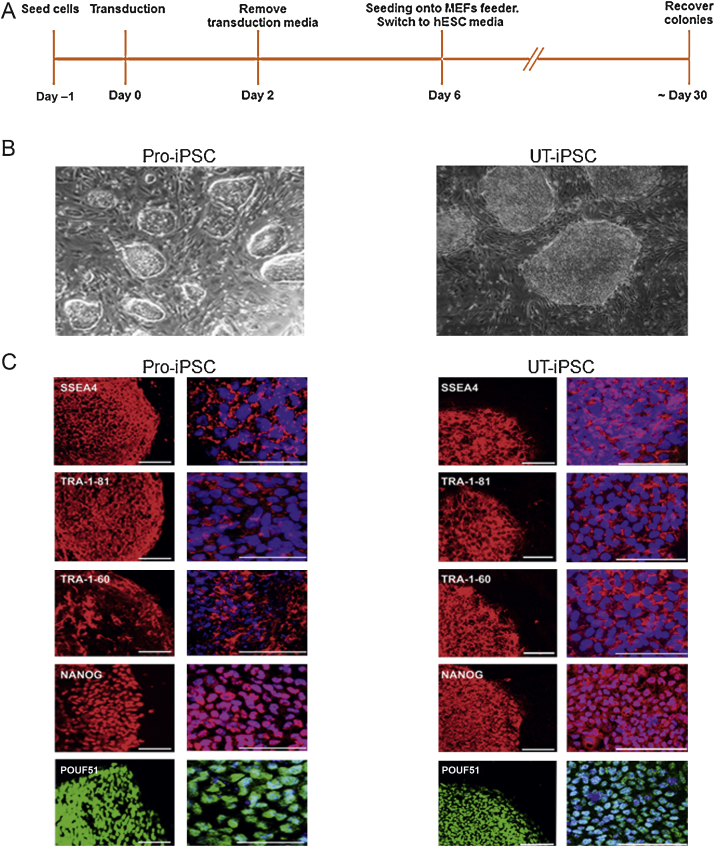
(A) Timeline for induced pluripotent stem cell (iPSC) generation. (B) Example of established iPSC colonies growing on a feeder layer with human embryonic stem cell (ESC)–like morphology. (C) Immunofluorescence of generated iPSCs for the expression of specific human ESC surface markers: stage-specific embryonic antigen-4 (SSEA4), tumour rejection antigen (TRA)-1-81, TRA-1-60, and nuclear transcription factors Nanog homeobox (NANOG) and POU class 5 homeobox 1(POU5F1, formerly OCT4). Note mouse embryonic fibroblast (MEF) cells at the periphery of the colonies are negative for stem cell marker expression. Nuclei were stained with 4′,6-diamidino-2-phenylindole (blue) (scale bar = 100 μm), and specific cellular localisation of the stem cell markers are shown in their expected distribution. hESC = human embryonic stem cell; Pro-iPSC = prostate induced pluripotent stem cell; UT-iPSC = urinary tract induced pluripotent stem cell.

Once primary cultures of prostate, bladder, and ureter stromal cells were established, we examined the purity of fibroblast lineage. Following passage, there was an inherent culture-based selection for fibroblasts based on cell morphology and lineage-marker expressions. Morphologically, cells were consistent with primary, prostate stromal fibroblasts and were confirmed to be devoid of endothelial, epithelial, and haematopoietic contamination following the first passage (Supplemental Fig. S1A and S1B). Pure bladder and ureter stromal cells expressing smooth muscle and myofibroblastic markers α-SMA and CD90 were confirmed at second passage, associated with typical stromal-cell appearance (Supplemental Fig. S2A and S2B). Polycistronic lentiviral vectors containing the four transcription factors POU5F1, SOX2, KLF4, and MYC were transduced into pure stromal cells. A schedule for human iPSC reprogramming is summarised in Figure 1A. Seven days following lentiviral transduction, fibroblasts demonstrated features of mesenchymal to epithelial transition, which is typical of early reprogramming [20]. Mesenchymal to epithelial transition was characterised by a change in the prostate fibroblast morphology from spindle-shaped cells to classic, epitheloid, cobblestone colonies, and was confirmed by marker expression showing upregulation of epithelial marker E-cadherin and downregulation of mesenchymal markers snail homolog 1 and 2 (Drosophila) (dubbed Snail and Slug, respectively) (*p* < 0.05) (Supplemental Fig. S1C). Four week to 5 wk after transduction with the lentivirus, several small and tight cell colonies were detected; however, they grew slowly and so were not consistent with ESC-like cells (Supplemental Fig. S2C). By week 6 after transduction, rapidly growing colonies displaying morphology similar to that of human ESCs were observed (tight and flat colonies with clear-cut edges composed of small cells with a high nucleus-to-cytoplasm ratio) (Fig. 1B, Supplemental Fig. S1D and S2D). Eleven prostate and 31 urinary tract ESC-like colonies (17 bladder and 14 ureter) were successfully expanded and stably maintained throughout culture passages (>50 passages, >10 mo). The overall efficiency in generating Pro-iPSCs and UT-iPSCs was low (0.02–0.04% of all stromal cells transfected), but was comparable to the reported efficiency of iPSCs generated from human dermal fibroblasts [21]. Karyotyping confirmed a diploid 46,XY chromosome arrangement (Supplemental Fig. S1E and S2E). Authentication of Pro-iPSCs and UT-iPSCs derivation from parental stromal cells was confirmed using DNA fingerprinting (Supplemental Fig. S1F and S2F).

### 3.2 Characterisation of generated induced pluripotent stem cells

**Fig. 2:**
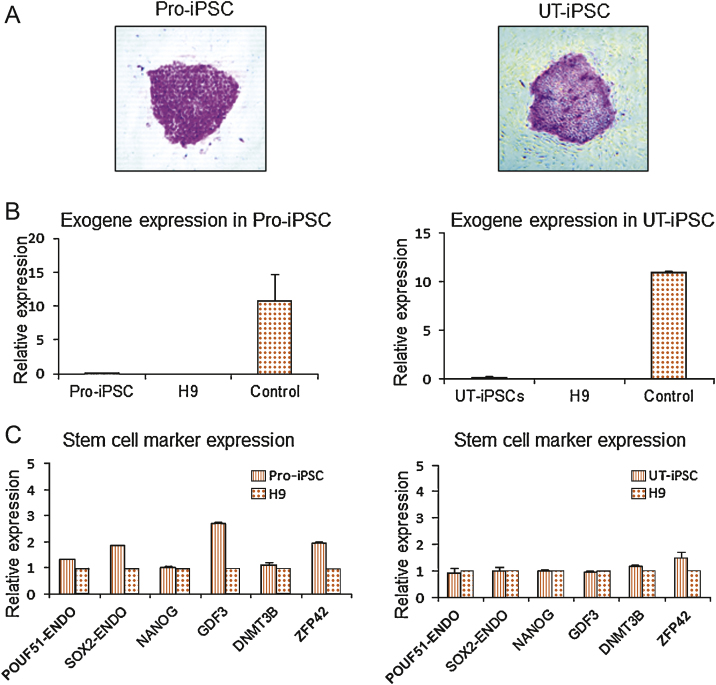
(A) Alkaline phosphatase staining of induced pluripotent stem cell (iPSC) colonies. Note underlying mouse embryonic fibroblast cells act as negative control cells with no staining. (B) Reverse transcription–polymerase chain reaction (RT-PCR) analysis demonstrating silencing of exogenous transgene expression in iPSCs. Control represents stromal cells 6 d after transduction. (C) Real-time RT-PCR analysis for expression of endogenous POU class 5 homeobox 1 (*POU5F1*, formerly OCT4), SRY (sex determining region Y)-box 2 (*SOX2*), and other stem cell marker genes: Nanog homeobox (*NANOG*), growth differentiation factor 3 (*GDF3*), DNA (cytosine-5-)-methyltransferase 3 beta (*DNMT3B*) and ZFP42 zinc finger protein (*ZFP42*, formerly REX1) in iPSCs. All values were calculated with respect to the value for H9 human embryonic stem cell, which was set to 1. Pro-iPSC = prostate induced pluripotent stem cell; UT-iPSC = urinary tract induced pluripotent stem cell; ENDO = endothelial.

Immunofluorescence for human, ESC-specific, surface markers—stage-specific embryonic antigen-4, tumour rejection antigen (TRA)-1-81, and TRA-1-60, and also transcription factors Nanog homeobox (*NANOG*) and *POU5F1*—was confirmed (Fig. 1C). Additionally, alkaline phosphatase activity, typical of an ESC phenotype, was demonstrated in the induced cells (Fig. 2A). Exogenous transgene silencing is associated with the generation of iPSCs, where there is a critical switch to endogenous expression of key ESC regulatory factors such as *POU5F1*, *SOX2*, and *NANOG*. Real-time RT-PCR using primers specific for lentiviral transcripts demonstrated that exogenous transgene expression had ceased in both Pro-iPSC and UT-iPSC clones (Fig. 2B). Furthermore, endogenous expression of the pluripotency markers *POU5F1*, *SOX2*, and *NANOG*, in addition to ESC markers growth differentiation factor 3 (*GDF3*), DNA (cytosine-5-)-methyltransferase 3 beta (*DNMT3B*), and ZFP42 zinc finger protein (*ZFP42*, formerly REX1) in both Pro-iPSCs and UT-iPSCs was consistent with expression levels in the control human-ESC line H9 (Fig. 2C). The iPSC clones were identical in terms of ESC-like morphology, proliferation, and gene-expression signatures (data not shown).

### 3.3 Pluripotency of generated induced pluripotent stem cells

**Fig. 3:**
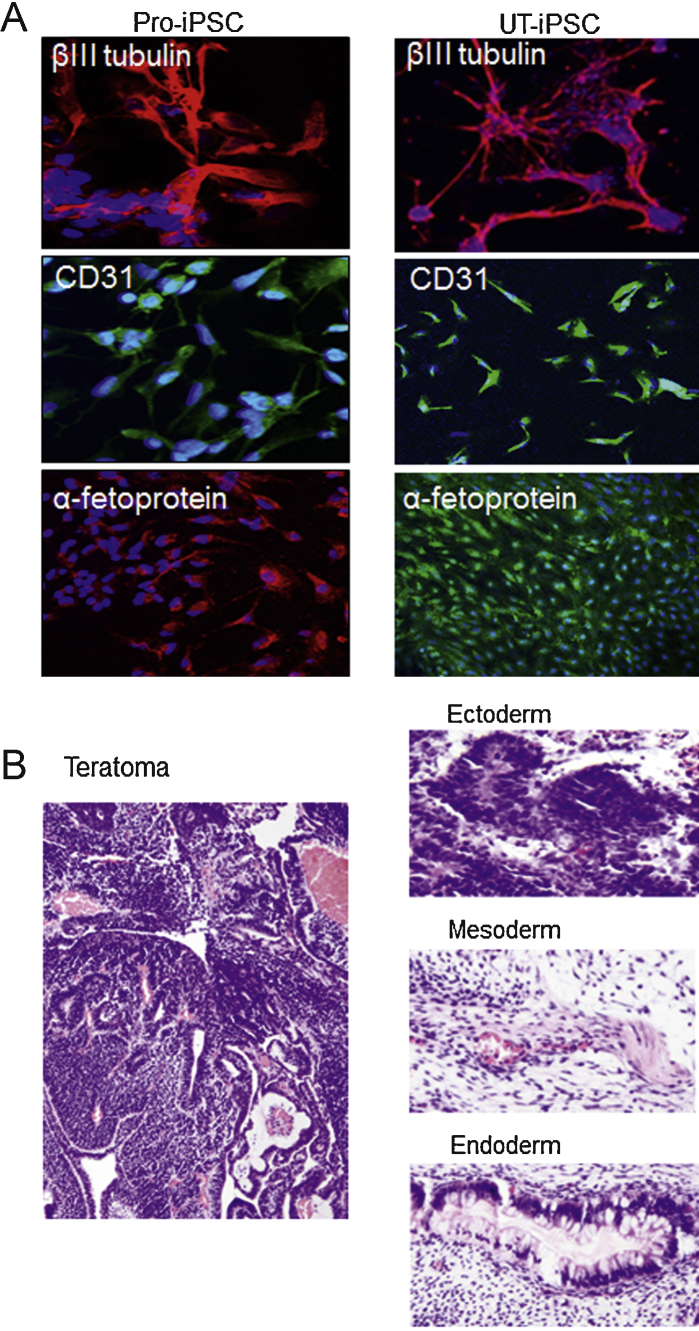
Pluripotency in prostate induced pluripotent stem cells (Pro-iPSCs) and urinary tract induced pluripotent stem cells (UT-iPSCs). (A) Immunofluorescence analysis of embyroid bodies derived from iPSCs shows expression of the lineage markers βIII-tubulin (ectodermal marker; red), CD31 (mesodermal marker; green), and α-fetoprotein (endodermal marker). Nuclei were counterstained with 4′,6-diamidino-2-phenylindole (blue) (scale bar = 100 μm). (B) Left: Histologic section of teratoma formed from Pro-iPSCs showing neuronal epithelial differentiation. Right: UT-iPSCs representing all three embryonic germ layers: ectoderm (neuronal rosette-like structures), mesoderm (muscle-like tissue), and endoderm (intestinal epithelial-like cells).

When cultured in suspension on low-adhesion plates in the absence of basic FGF, both Pro-iPSCs and UT-iPSCs formed embryoid bodies containing all three germ-layer derivatives, as demonstrated by immunofluorescence of lineage-specific markers βIII tubulin (ectoderm), CD31 (mesoderm), and α-fetoprotein (endoderm) (Fig. 3A). Xenografts from the in vivo teratoma-forming assay also confirmed ectoderm, mesoderm, and endoderm lineage histology (Fig. 3B).

### 3.4 Prostate induced pluripotent stem cells differentiate into androgen receptor and prostate-specific antigen–expressing cells

**Fig. 4:**
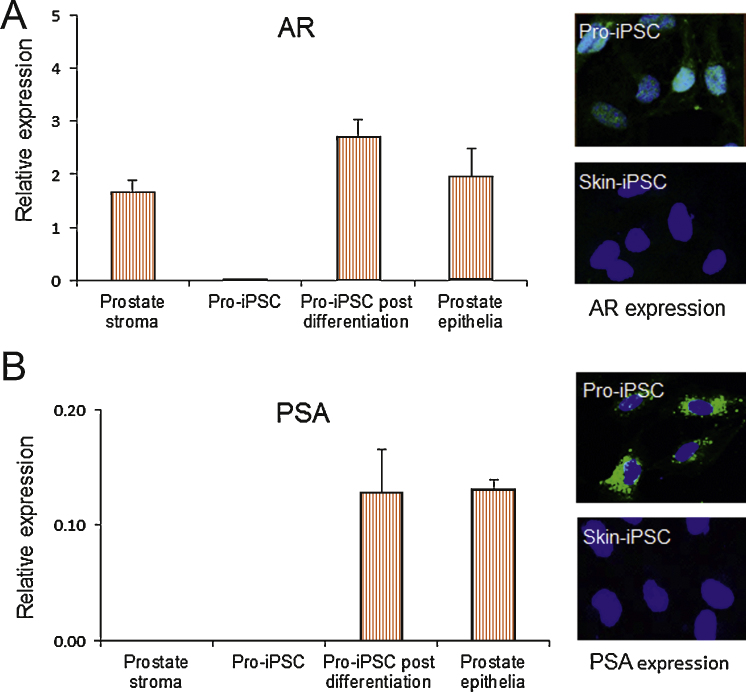
Prostate-specific differentiation of prostate induced pluripotent stem cells (Pro-iPSCs). (A) Relative messenger RNA (mRNA) expression of androgen receptor (AR) in Pro-iPSCs before and after differentiation (control levels in primary prostate stroma and epithelia shown) (*n* = 6). Right: Immunofluorescence staining for AR (green) shown in Pro-iPSCs and skin-iPSCs. AR is induced in Pro-iPSCs only, with functional activity suggested by nuclear AR localisations (blue 4′,6-diamidino-2-phenylindole [DAPI] counterstain). (B) Relative mRNA expression of prostate-specific antigen (PSA) in Pro-iPSCs before and after differentiation (control levels in primary prostate stroma and epithelia shown) (*n* = 3). Immunofluorescence staining for PSA (green) shown in Pro-iPSCs and skin-iPSCs. PSA is induced in Pro-iPSCs only (blue DAPI counterstain).

We compared the differentiation potential of Pro-iPSCs with conventional skin-derived iPSCs (skin-iPSCs). Following induced differentiation by prostate stromal-cell conditioned medium for 3 wk, both Pro-iPSCs and skin-iPSCs displayed epithelioid cell morphology. Transcripts of epithelial marker CD24 were detected at comparable levels to primary prostate epithelium (data not shown). When characterised with the prostate differentiation markers androgen receptor (AR) and prostate-specific antigen (PSA), the prostate-specific phenotype was restricted to Pro-iPSCs (Fig. 4). AR expression was present in the differentiated Pro-iPSCs and the degree of immunofluorescence was at levels comparable with mature prostate epithelium in early primary culture (day 10), whereas only minimal levels of AR were detected in skin-iPSCs (Fig. 4A). Functional AR was confirmed by PSA transcript and protein expression at levels in keeping with primary, cultured, prostate epithelia, whereas no PSA expression was detected in the differentiation medium-treated skin-iPSCs (Fig. 4B).

### 3.5 Urinary tract-induced pluripotent stem cells differentiate into uroplakin-expressing cells

**Fig. 5:**
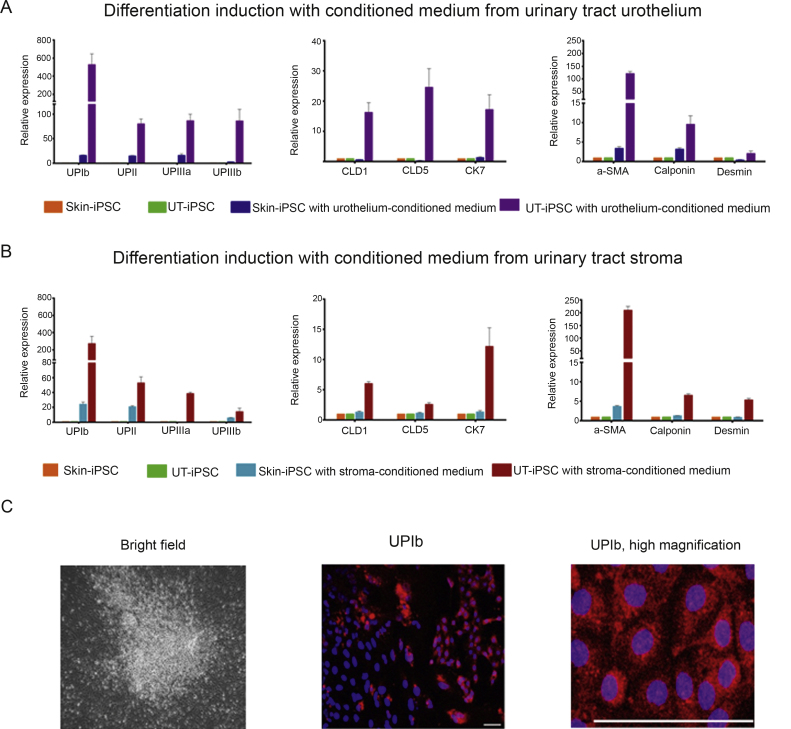
Expression of urothelial and smooth-muscle lineage-specific transcripts in differentiated cells derived from urinary tract induced pluripotent stem cells (UT-iPSCs) and skin-iPSCs by reverse transcription-polymerase chain reaction on day 14. Messenger RNA levels are shown as a fold change relative to control (undifferentiated cells) (*n* = 3). (A) Expression of urothelial-specific markers (UPIb, UPII, UPIIIa, UPIIIb, claudin 1 [*CLD1*], claudin 5 [*CLD5*], keratin 7 [*CK7*]) and smooth-muscle-specific markers (α-smooth muscle actin [a-SMA], calponin, desmin) induced with conditioned medium from urothelium. (B) Expression of urothelial- and smooth-muscle–specific markers induced with conditioned medium from stroma. (C) Immunofluorescence of differentiated cells derived from UT-iPSCs treated with conditioned medium, at day 14, showing (left) bright field; (centre) positive staining for UPIb (red) juxtaposed with an area of UPIb-negative staining, with 4′,6-diamidino-2-phenylindole nuclear counterstain (blue); and high magnification of UPIb immunostaining (scale bar = 100 μm).

Lineage-specific differentiation of the UT-iPSCs into bladder lineages was assessed using established coculture methods [18]. In comparison to cells derived from skin-iPSCs, UT-iPSCs were significantly more efficient at inducing bladder-specific differentiation, as demonstrated by urothelial differentiation-specific genes (UPIb, UPII, UPIIIa, and UPIIIb; claudin 1 [CLD1], claudin 5 [CLD5] and keratin 7 [CK7]) and markers specific for smooth-muscle cells (α-SMA, calponin, and desmin) (Fig. 5A). Furthermore, the effect of conditioned medium from urothelia was compared with that of conditioned medium from stromal cells (Fig. 5B). Similar to the embryogenesis of the urinary tract, where there is a reciprocal differentiation of epithelial and mesenchymal fractions [22], both urothelial and stromal differentiation was concurrently induced using either urothelial- or stromal-conditioned medium (Fig. 5A and 5B). UPIb protein expression indicated by immunofluorescence was also confirmed in differentiated cells derived from UT-iPSCs after 2 wk in urothelial-conditioned medium (Fig. 5C).

## 4 Discussion

We report for the first time successful reprogramming of normal, human, ageing prostate, bladder, and ureter stromal fibroblasts to an ESC-like pluripotent state. These cells were validated as de facto iPSCs by confirming their ability for sustained self renewal, silencing of exogenous transgenes, expression of ESC-specific genes, and pluripotent differentiation into all three germ lineages. Furthermore, within the appropriate inductive environment, prostate epithelial-specific differentiation with induced functional AR, as characterised by PSA expression, was demonstrated and UT-iPSC differentiation could be directed into urothelial-specific lineages. These models allow for enormous scope in future studies of differentiation, tissue engineering, disease mechanisms, and drug development.

Since the first descriptions of iPSCs in 2006, most reports have focused on the generation of iPSCs from a range of normal and diseased tissues. In our work, we generated multiple clones of UT-iPSCs from multiple donors and sites (bladder and ureter), and multiple clones of Pro-iPSCs from a single donor. All clones were comparable to both skin-iPSCs and ESCs in terms of ESC-marker expression and pluripotency potential, supporting the generalisability of these methods to urologic tissues. Researchers exploring further differences in ability for terminal organ-specific differentiation among the iPSC lines derived from different organs have found that not all iPSCs are the same in this respect [13]. We compared the differentiation potential of Pro-iPSCs and UT-iPSCs with skin-iPSCs and our results demonstrated vast differences in their capabilities for prostate- and bladder-specific differentiation. These findings are consistent with emerging evidence that epigenetic imprinting, specific to the tissue type of iPSC origin, remains intact throughout the mechanism of stem cell reprogramming and may limit the potential for terminal differentiation into all cell types [12–14]. Our data emphasise the importance of the source from which iPSCs are generated as a consideration for organ-specific development studies.

Although we confirmed prostate-specific differentiation, additional work is required to determine whether Pro-iPSCs can generate the full breadth of epithelial differentiation. In addition, it would be of interest to investigate if these cells differentiate through somatic stem cell phenotypes or directly into basal and luminal cells, in keeping with an alternative model that proposes that epithelial stem cells maintain the phenotype of the original embryonic progenitor of the prostate (urogenital sinus epithelium) [23]. Also of interest would be further in vivo study of prostate epithelial development and organisation from iPSCs, especially given the considerable debate about the nature of the somatic stem cell in prostate epithelium. Recent evidence has shown that in early, postnatal, mouse prostate development, epithelial homeostasis is maintained by basal multipotent stem cells that differentiate into basal, luminal, and neuroendocrine cells, as well as by unipotent basal and luminal progenitors [24]. In contrast, in situ assays in the human adult setting have revealed that epithelial hierarchical organisation is based on a common stem cell [25]. The Pro-iPSC model is well placed to explore these differences with lineage tracking studies. Furthermore, the generation of ESC-like Pro-iPSCs are of particular interest to the study of prostate disease, where benign prostatic hyperplasia has been associated with models of embryonic reawakening [26] and prostate cancer can be associated with ESC-marker expressions [27].

Atala and colleagues reported the first human clinical trial with engraftment of both urothelial and smooth-muscle stromal cells into acellular biomaterials for bladder engineering and reconstruction [28]. However, these strategies rely on ex vivo cell culture to generate sufficient quantities and quality of autologous cells. Although even small biopsies of normal urothelium can be readily expanded before undergoing senescence, this is significantly restricted in diseased tissue [29]. We were unsuccessful in preliminary attempts at inducing urothelium. Further experimentation was not pursued given that the stroma-based UT-iPSCs demonstrated ability to differentiate into urothelium. In clinical applications for regenerative medicine, this phenomenon also provides the additional attraction of using an alternative, genetically normal tissue source in urothelial malignancies for reconstruction. However, concerns about induction of malignancies from iPSCs persist and are being cautiously tackled with refinements in induction methods that include virus-free and transgene-free reprogramming and xeno-free approaches [30]. Alternative approaches are now becoming established to realise this ambition, including vector-free human-iPSC generation using episomal-factor delivery, and feeder-free and albumin-free culture [31]. As such, UT-iPSCs show great promise in clinical regenerative medicine and modelling urinary tract disease.

## 5 Conclusions

Human prostate and urinary tract tissue can be used to generate iPSCs that can be differentiated back into their parent organ lineages. The generation of Pro-iPSCs and UT-iPSCs provides a convenient ready-to-access model that offers considerable potential for studies of normal and diseased prostate and bladder biology.

  ***Author contributions:*** Craig Robson had full access to all the data in the study and takes responsibility for the integrity of the data and the accuracy of the data analysis.  

*Study concept and design:* Robson, Heer.

*Acquisition of data:* Moad, Pal, Hepburn, Williamson, Franco, Fordham.

*Analysis and interpretation of data:* Robson, Heer, Moad, Pal, Hepburn, Williamson, Hayward, Franco, Lako, Armstrong, Fordham, Przyborski.

*Drafting of the manuscript:* Robson, Heer, Pal, Moad.

*Critical revision of the manuscript for important intellectual content:* Moad, Pal, Hepburn, Williamson, Wilson, Lako, Armstrong, Hayward, Franco, Cates, Fordham, Przyborski, Carr-Wilkinson, Robson, Heer.

*Statistical analysis:* Moad.

*Obtaining funding:* Robson, Heer.

*Administrative, technical, or material support:* Robson, Heer.

*Supervision:* Robson, Heer.

*Other* (specify): None.  

***Financial disclosures:*** Craig Robson certifies that all conflicts of interest, including specific financial interests and relationships and affiliations relevant to the subject matter or materials discussed in the manuscript (eg, employment/affiliation, grants or funding, consultancies, honoraria, stock ownership or options, expert testimony, royalties, or patents filed, received, or pending), are the following: None.  

***Funding/Support and role of the sponsor:*** This work was supported by grants from the JGWP Foundation, Cancer Research UK, EU FP7 Marie Curie Integrated Training Network (PRO-NEST), and the NIH (R01 DK067049). Cancer Research UK, PRO-NEST, Newcastle University, was involved in the design and conduct of the study.

## Figures and Tables

**Table 1:** Details of patients from whom induced pluripotent stem cells lines were established

Patent identifier	Age, yr	Sex	Nature of tissue biopsy
12380	65	Female	Ureter biopsy from radical nephrectomy for renal cell carcinoma
12459	66	Male	Bladder biopsy from cystoprostatectomy undertaken as part of urinary diversion for benign functional disorder (secondary to urethral injury and bowel surgery)
12491	48	Male	Bladder biopsy from cystoprostatectomy for benign, functional, neurologic disorder
12502	54	Male	Ureter biopsy from radical nephrectomy for renal cell carcinoma
12506	56	Male	Bladder biopsy from cystoprostatectomy for benign functional neurologic disorder
11901	66	Male	Prostate biopsy from cystoprostatectomy for bladder cancer

## References

[1] Cunha G.R. (1994). Role of mesenchymal-epithelial interactions in normal and abnormal development of the mammary gland and prostate. Cancer.

[2] Cunha G.R., Hayward S.W., Wang Y.Z. (2002). Role of stroma in carcinogenesis of the prostate. Differentiation.

[3] Staack A., Hayward S.W., Baskin L.S., Cunha G.R. (2005). Molecular, cellular and developmental biology of urothelium as a basis of bladder regeneration. Differentiation.

[4] Kolli S., Lako M., Figueiredo F., Mudhar H., Ahmad S. (2008). Loss of corneal epithelial stem cell properties in outgrowths from human limbal explants cultured on intact amniotic membrane. Regen Med.

[5] Birgersdotter A., Sandberg R., Ernberg I. (2005). Gene expression perturbation in vitro–a growing case for three-dimensional (3D) culture systems. Semin Cancer Biol.

[6] Ross D.T., Scherf U., Eisen M.B. (2000). Systematic variation in gene expression patterns in human cancer cell lines. Nat Genet.

[7] Oottamasathien S., Wang Y., Williams K. (2007). Directed differentiation of embryonic stem cells into bladder tissue. Dev Biol.

[8] Taylor R.A., Cowin P.A., Cunha G.R. (2006). Formation of human prostate tissue from embryonic stem cells. Nat Methods.

[9] Takahashi K., Yamanaka S. (2006). Induction of pluripotent stem cells from mouse embryonic and adult fibroblast cultures by defined factors. Cell.

[10] Yu J., Vodyanik M.A., Smuga-Otto K. (2007). Induced pluripotent stem cell lines derived from human somatic cells. Science.

[11] Robinton D.A., Daley G.Q. (2012). The promise of induced pluripotent stem cells in research and therapy. Nature.

[12] Kim K., Doi A., Wen B. (2010). Epigenetic memory in induced pluripotent stem cells. Nature.

[13] Polo J.M., Liu S., Figueroa M.E. (2010). Cell type of origin influences the molecular and functional properties of mouse induced pluripotent stem cells. Nat Biotechnol.

[14] Lister R., Pelizzola M., Kida Y.S. (2011). Hotspots of aberrant epigenomic reprogramming in human induced pluripotent stem cells. Nature.

[15] Heer R., Robson C.N., Shenton B.K., Leung H.Y. (2007). The role of androgen in determining differentiation and regulation of androgen receptor expression in the human prostatic epithelium transient amplifying population. J Cell Physiol.

[16] Williamson S.C., Hepburn A.C., Wilson L. (2012). Human alpha(2)beta(1)(HI) CD133(+VE) epithelial prostate stem cells express low levels of active androgen receptor. PloS One.

[17] Southgate J., Hutton K.A., Thomas D.F., Trejdosiewicz L.K. (1994). Normal human urothelial cells in vitro: proliferation and induction of stratification. Lab Invest.

[18] Tian H., Bharadwaj S., Liu Y., Ma P.X., Atala A., Zhang Y. (2010). Differentiation of human bone marrow mesenchymal stem cells into bladder cells: potential for urological tissue engineering. Tissue Eng Part A.

[19] Lowry W.E., Richter L., Yachechko R. (2008). Generation of human induced pluripotent stem cells from dermal fibroblasts. Proc Natl Acad Sci U S A.

[20] Li R., Liang J., Ni S. (2010). A mesenchymal-to-epithelial transition initiates and is required for the nuclear reprogramming of mouse fibroblasts. Cell Stem Cell.

[21] Takahashi K., Tanabe K., Ohnuki M. (2007). Induction of pluripotent stem cells from adult human fibroblasts by defined factors. Cell.

[22] Baskin L.S., Hayward S.W., Young P., Cunha G.R. (1996). Role of mesenchymal-epithelial interactions in normal bladder development. J Urol.

[23] Wang Y., Hayward S., Cao M., Thayer K., Cunha G. (2001). Cell differentiation lineage in the prostate. Differentiation.

[24] Ousset M., Van Keymeulen A., Bouvencourt G. (2012). Multipotent and unipotent progenitors contribute to prostate postnatal development. Nat Cell Biol.

[25] Blackwood J.K., Williamson S.C., Greaves L.C. (2011). In situ lineage tracking of human prostatic epithelial stem cell fate reveals a common clonal origin for basal and luminal cells. J Pathol.

[26] Isaacs J.T., Coffey D.S. (1989). Etiology and disease process of benign prostatic hyperplasia. Prostate Suppl.

[27] Glinsky G.V. (2008). “Stemness” genomics law governs clinical behavior of human cancer: implications for decision making in disease management. J Clin Oncol.

[28] Atala A., Bauer S.B., Soker S., Yoo J.J., Retik A.B. (2006). Tissue-engineered autologous bladders for patients needing cystoplasty. Lancet.

[29] Subramaniam R., Hinley J., Stahlschmidt J., Southgate J. (2011). Tissue engineering potential of urothelial cells from diseased bladders. J Urol.

[30] Warren L., Manos P.D., Ahfeldt T. (2010). Highly efficient reprogramming to pluripotency and directed differentiation of human cells with synthetic modified mRNA. Cell Stem Cell.

[31] Chen G., Gulbranson D.R., Hou Z. (2011). Chemically defined conditions for human iPSC derivation and culture. Nat Methods.

