# Supplementary material for: A Novel Model of Urinary Tract Differentiation, Tissue Regeneration, and Disease: Reprogramming Human Prostate and Bladder Cells into Induced Pluripotent Stem Cells
Source: Eur Urol. 2013 Nov;64(5):753–61. doi: 10.1016/j.eururo.2013.03.054 (PMC3819995; doi:10.1016/j.eururo.2013.03.054)
Supplement: Supplementary file 1 [file mmc1.pdf]

Figure S1

A

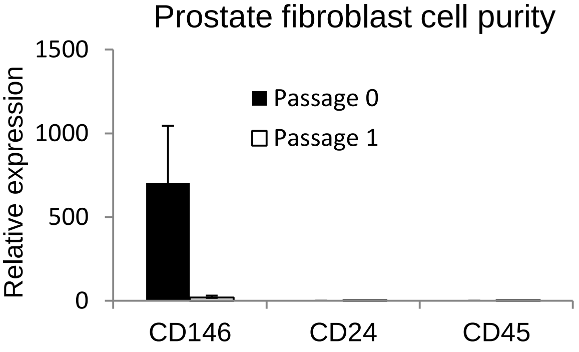

B

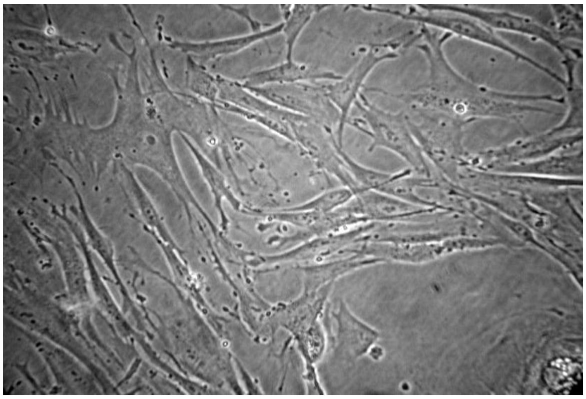

C

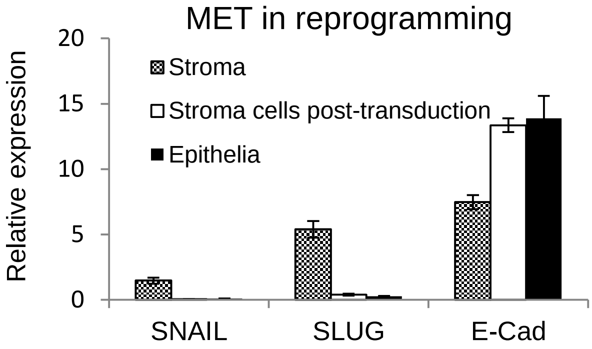

D

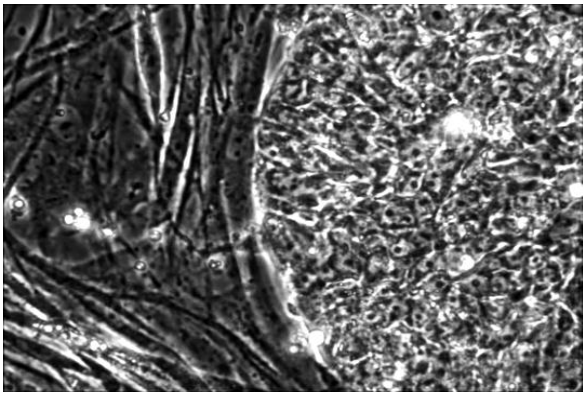

E

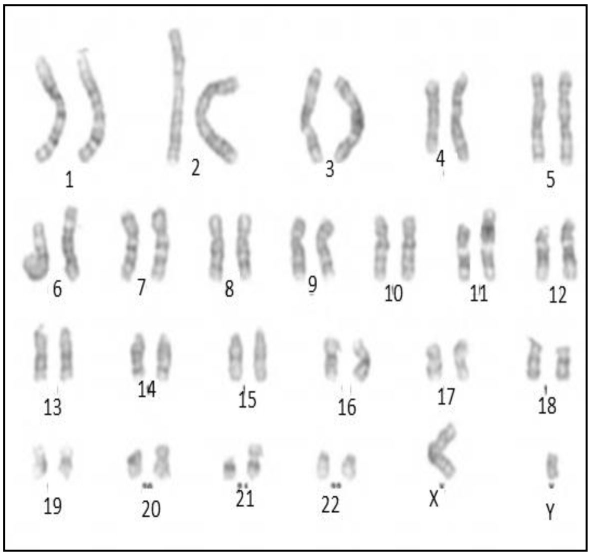

F

Marker	Pro-iPSCs	Parent fibroblast
Amelogenin	XY	XY
THO1	9.3 - 9.3	9.3 - 9.3
D21S11	30 - 31.2	30 - 31.2
D18S51	15 - 17	15 - 17
D13S317	8 - 12	8 - 12
D7S820	9 - 11	9 - 11
D16S539	11 - 13	11 - 13
PentaD	9 - 11	9 - 11
D8S1179	13 - 14	13 - 14
TPOX	8 - 11	8 - 11
FGA	21 - 25	21 - 25

Figure S1: iPSC generation from the human primary prostate. **A:** Prostate primary cultured fibroblasts after first passage compared with before culture (passage 0), confirm an absence of contamination from endothelia (CD146), epithelia (CD24) and blood cells (CD45). **B:** Primary prostate stroma cultures showing characteristic fibroblastic morphology. **C:** At day 7, mesenchymal to epithelial transition (MET) was demonstrated by down-regulation of snail and slug and up-regulation of E-cadherin transcripts. Hashed bars represent prostate stroma before transduction, blank bars represent prostate stroma after transduction and black bars represent corresponding levels in matched prostate epithelia **D:** Pro-iPSC colony at X40 magnifications with well-defined border containing a cluster of very tightly packed cells. **E:** Pro-iPSC confirmed to possess a diploid 46XY karyotype. **F:** DNA fingerprinting showing identical microsatellite genotyping between Pro-iPSCs and parent prostate cells.

Figure S2

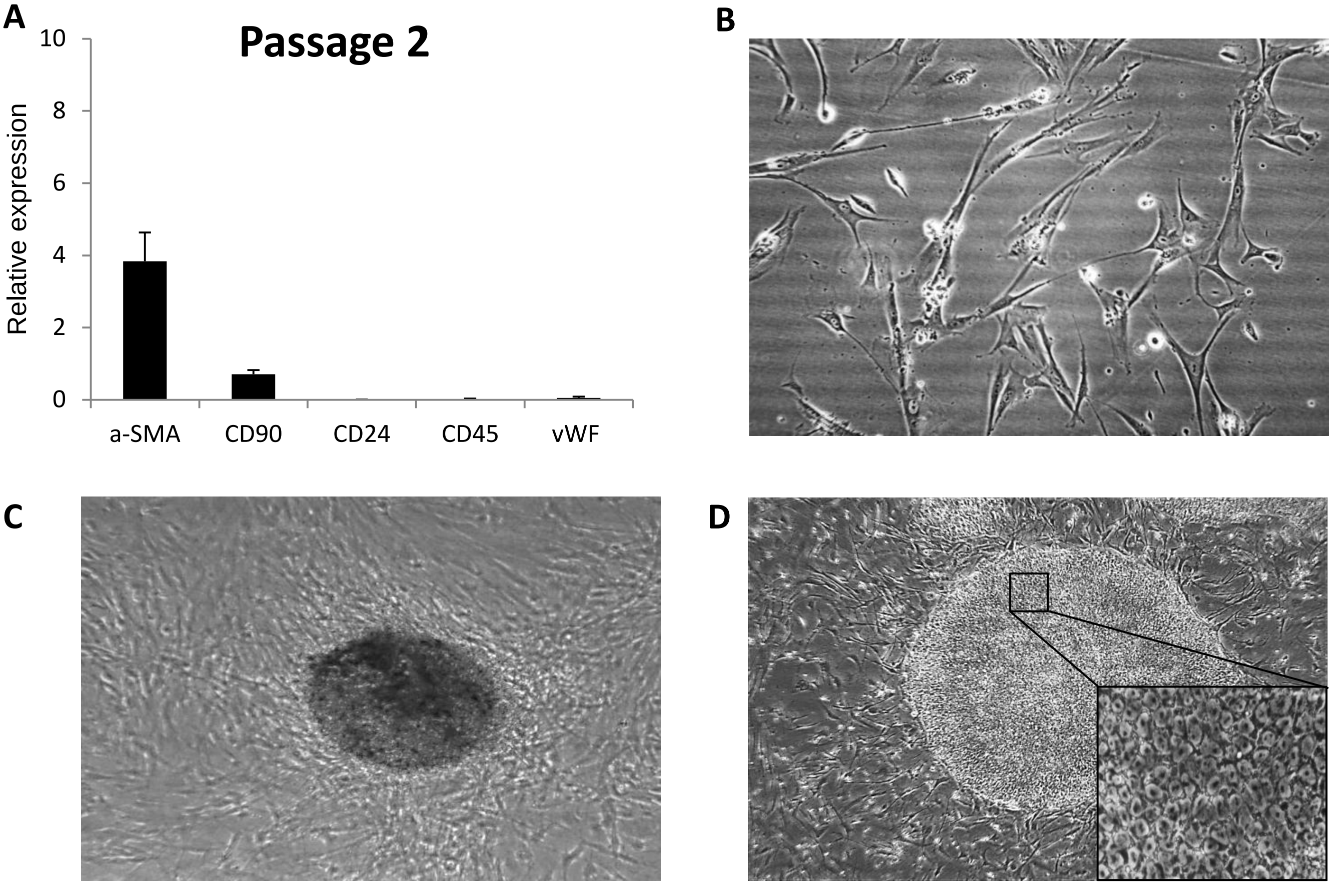

E

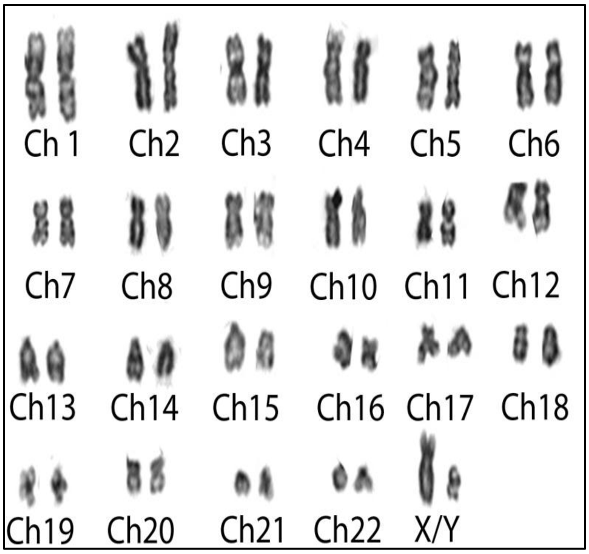

F

Marker	Parent fibroblast	UT-iPSCs
**Amelogenin**	**XY**	**XY**
**THO1**	**6 9.3**	**6 9.3**
**D21S11**	**29 30**	**29 30**
**D18S51**	**13 15**	**13 15**
**D7S829**	**8 9**	**8 9**
**D16S539**	**10 13**	**10 13**
**PentaD**	**9 10**	**9 10**
**D8S1179**	**12 12**	**12 12**
**TPOX**	**8 10**	**8 10**
**FGA**	**21 24**	**21 24**

Figure S2: iPSC generation from the human urinary tract cells. **A:** Real time-RT-PCR for mRNA expression of α-SMA, CD90 (stromal cell marker), CD24 (epithelial cell marker), CD45 (haematopoietic cell marker), and vWF (endothelial cell markers) in urothelial-associated stromal cells at Passage 2. Error bars show SEM for n=3. **B:** bright-field image of parental human stromal cells. **C:** Example of small and tight cell colonies observed 4 weeks after transduction. **D**: Example of established UT-iPSCs colonies growing on a feeder layer with human ES-cell-like morphology (P6). Insert shows higher magnification of boxed areas. **E:** Karyotype analysis shows normal karyotype of established UT-iPSCs at passage 20. **F:** DNA fingerprinting shows identical DNA genetic profiles between UT-iPSCs and matched parental stroma.
