# Supplementary material for: A Novel Model of Urinary Tract Differentiation, Tissue Regeneration, and Disease: Reprogramming Human Prostate and Bladder Cells into Induced Pluripotent Stem Cells
Source: Eur Urol. 2013 Nov;64(5):753–61. doi: 10.1016/j.eururo.2013.03.054 (PMC3819995; doi:10.1016/j.eururo.2013.03.054)
Supplement: Supplementary file 2 [file mmc2.doc]

**Supplement 1 – Protocols for cell culture**

***Prostate cell culture***

Prostate biopsy specimens were cut into 1-mm cubes and digested overnight for 12 h in 250 U/mg collagenase Type I (Collagenase CLS-2; Worthington Biochemical Corp, Lakewood, NJ, USA) to release epithelial and stromal fractions. Prostate stromal cells were maintained in Roswell Park Memorial Institute (RPMI) 1640 medium supplemented (Sigma-Aldrich Co, St. Louis, MO, USA) with 5% foetal bovine serum, 2 mM l-glutamine, and 1% penicillin-streptomycin. Prostate epithelial cells were routinely cultured in Collagen-I-coated flasks (BD Biosciences, Franklin Lakes, NJ, USA) in serum-free, low-calcium-containing keratinocyte basal medium (KSFM; Invitrogen Corp, Carlsbad, CA, USA) supplemented with 25 mg bovine pituitary extract (Invitrogen Corp, Carlsbad, CA, USA) and 2.5 µg recombinant epidermal growth factor (Invitrogen Corp, Carlsbad, CA, USA). The medium was further supplemented with 100 ng/ml cholera toxin (Sigma-Aldrich Co, St. Louis, MO, USA), 0.2 ng/ml leukaemia inhibitory factor (Sigma-Aldrich Co, St. Louis, MO, USA), 0.1 ng/ml granulocyte-macrophage colony-stimulating factor (Sigma-Aldrich Co, St. Louis, MO, USA), 0.2 ng/ml stem cell factor (Sigma-Aldrich Co, St. Louis, MO, USA). The epithelial cells were routinely cultured on mitomycin-C-inactivated murine STO cells (mouse embryonic fibroblast-cell line).

***Bladder cell culture***

Bladder or ureteric biopsy specimens were dissected to release urothelium from underlying stroma. The stroma was then digested by incubating with 250 U/mg collagenase Type I (Collagenase CLS-2; Worthington Biochemical Corp, Lakewood, NJ, USA) overnight at 37°C with stirring. Stromal cells were propagated and maintained in RPMI 1640 medium (with HEPES modification) (RPMI 1640; Sigma-Aldrich Co, St. Louis, MO, USA) containing 10% foetal calf serum (FCS) (Sigma-Aldrich Co, St. Louis, MO, USA), l-glutamine (2 mM) and 1% penicillin and streptomycin (Invitrogen Corp, Carlsbad, CA, USA). Urothelial cells were propagated in KSFM supplemented with epidermal growth factor (5 ng/ml) and bovine pituitary extract (50 pg/ml) as provided by the manufacturer (Invitrogen Corp, Carlsbad, CA, USA), as well as 1% penicillin and streptomycin (Invitrogen Corp, Carlsbad, CA, USA), and 30 ng/ml cholera toxin (Sigma-Aldrich Co, St. Louis, MO, USA); added to improve cell attachment, this medium was called complete keratinocyte serum-free medium. The culture flask was maintained at cell culture conditions (37°C, 5% carbon dioxide) and the medium was replaced every other day.

***Optimisation of lentiviral transductions of stromal cells***

A green fluorescent protein–lentivirus construct was initially used to evaluate a range of multiplicity of infection (MOI) assessed by flow cytometry. In addition, we assessed the toxicity of varying polybrene concentrations before the optimised protocols, as described in the main text, were established. Of note, the effectiveness in generating induced pluripotent stem cell (iPSC) clones varied from batch to batch of the viral particles supplied, and this is best tackled by considering simultaneous parallel transductions of different viral batches when possible. Early colony formation (within 2 wk) can be misleading and, to the inexperienced eye, these can appear similar to embryonic stem cell (ESC) morphology but may, in fact, represent abortive colonies [1]. We found that delaying the selection of the best clones until 4 wk helped identify the de facto iPSCs.

***Lentivirus transduction of epithelial cells***

The protocol used for stroma cells was also applied to the primary culture of human urothelial and prostate epithelial cells that were maintained in KSFM. The induction protocol used was identical to that described for stroma induction. However, cells were not viable in most instances (*n* = 6), and when colonies were seen, these were nonproliferative and the morphology was not in keeping with ESCs (*n* = 6 for bladder cells and these cells were in keeping with the colonies demonstrated in Supplemental Fig. 2c). Further reduction of toxicity from optimisation of MOI, of polybrene, and of timing of transfer to ESC medium with mouse embryonic fibroblasts (MEFs) is required.

***RNA isolation, reverse transcription, and polymerase chain reaction***

Total RNA was purified using RNeasy Kit (Qiagen, Venlo, The Netherlands) according to manufacturer’s instructions and treated with DNase to remove genomic DNA contamination. Moloney murine leukaemia virus reverse transcription (M-MLV RT) (Promega Corp, Madison, WI, USA) was followed by quantitative polymerase chain reaction (PCR) using SYBR Green (Sigma-Aldrich Co, St. Louis, MO, USA), analysed using the ABI PRISM 7900 HT sequence detection system (Applied Biosystems/Invitrogen Corp, Carlsbad, CA, USA) and normalised to glyceraldehyde 3-phosphate dehydrogenase (GAPDH).

***Primers for real-time reverse transcription-polymerase chain reaction***

| Genes | Forward primer (5′−3′) | Reverse primer (5′−3′) |
| --- | --- | --- |
| *GAPDH* | CGACCACTTTGTCAAGCTCA | GGGTCTTACTCCTTGGAGGC |
| *CD24* | TGAAGAACATGTGAGAGGTTTG | GAAAACTGAATCTCCATTCCAC |
| *vWF* | ACTGAAGCGTGATGAGACGC | TTCATCAAAGGGTGGGCAGC |
| *CD45* | GAAATTGTTCCTCGTCTGAT | CTTTGCCCTGTCACAAATAC |
| α-SMA | CCGACCGAATGCAGAAGGA | ACAGAGTATTTGCGTCCGAA |
| *CD90* | CACACARACCGCTCCCGAACC | GCTGATGCCCTCACACTT |
| ENDO-*PAU5F1* | GCAAGCCCTCATTTCACCAGGCC | AGGATCAACCCAGCCCGGCT |
| ENDO-*SOX2* | TCACATGTCCCAGCACTACC | CCCATTTCCCTCGTTTTTCT |
| *NANOG* | CCAAATTCTCCTGCCAGTGAC | CACGTGGTTTCCAAACAAGAAA |
| *GDF3* | CTTATGCTACGTAAAGGAGCTGGG | GTGCCAACCCAGGTCCCGGAAGTT |
| *ZFP42* (formerly REX1) | CGTACGCAAATTAAAGTCCAGA | CAGCATCCTAAACAGCTCGCAGAAT |
| *DNMT3B* | TGCTGCTCACAGGGCCCGATACTTC | TCCTTTCGAGCTCAGTGCACCACAAAAC |
| *AR* | CTGGACACGACAACAACCAG | CAGATCAGGGGCGAAGTAGA |
| *KLK3* (PSA) | CAATGACGTGTGTGCGCAA | CGTGATACCTTGAAGCACACCA |
| UPIb | GGGACAGACAAGGTGCCTGTTAT | TATTGGCTGGCTTGCTTCTCTCCA |
| UPII | CAGTGCCTCACCTTCCAACA | TGGTAAAATGGGAGGAAAGTCAA |
| UPIIIa | TCACTGGCACCCACGAGGTCT | CGTTGAGCCCAGTGGGGTGTT |
| UPIIIb | CCCTGGCCCTGGACCCTATCG | CCACAGGCTGGAGAAGCGCA |
| Calponin | TTTGAGGCCAACGACCTGTT | CCTTTCGTCTTCGCCATGCT |
| Desmin | CCATCGCGGCTAAGAACATT | TCGGAAGTTGAGGGCAGAGTA |
| *CLDN1* | ATGGAAAGGGTGTTGGCATTGGTG | AATGCCTTGCTCAAACACAGACGG |
| *CLDN5* | CTGTTTCCATAGGCAGAGCG | AAGCAGATTCTTAGCCTTCC |
| *CK7* | TGTGGTGCTGAAGAAGGATGTGGA | TGTCAACTCCGTCTCATTGAGGGT |
| Transgene | TGCTGCCAAGAGGGTCAAG | AGCCATACGGGAAGCAATAG |

GAPDH = glyceraldehyde 3-phosphate dehydrogenase; vWF = von Willebrand factor; SMA = smooth muscle actin; CD90 = Thy-1 cell surface antigen; ENDO = endothelial; POU5F1 = POU class 5 homeobox 1 (formerly OCT4); SOX2 = SRY (sex determining region Y)-box 2; NANOG = Nanog homeobox; GDF3 = growth differentiation factor 3; ZFP42 = zinc finger protein (formerly REX1); DNMT3B = DNA (cytosine-5-)-methyltransferase 3 beta; AR = androgen receptor; KLK3 = kallikrein-related peptidase 3; PSA = prostate-specific antigen; CLDN1 = claudin 1; CLDN5 = claudin 5; CK7 = keratin 7.

***DNA fingerprinting and karyotyping***

Total genomic DNA of generated iPSCs and parent stromal cells was extracted and amplified with microsatellite markers for a short tandem repeat assay and analysed on an ABI377 sequence detector using Genotype software (Applied Biosystems/Invitrogen Corp, Carlsbad, CA, USA). The karyotype of iPSCs was determined by metaphase arrest through colchicine treatment, Giemsa banding using Giemsa staining and Kario software (Delta Sistemi, Rome, Italy).

***Immunofluorescence and alkaline phosphatase staining***

An alkaline phosphatase detection kit was used according to manufacturer’s instructions with fast red violet, AS-BI phosphate solution and distilled water at a 2:1:1 ratio for 15 min (Chemicon, Temecula, CA, USA). For immunofluorescence, cells were fixed in 4% paraformaldehyde and permeabilised with 0.1% Triton X-100. Primary antibodies included anti-OCT4 (1:100; Millipore Corp, Billerica, MA, USA; CN.MAB4401A4); anti-NANOG (1:100; Cell Signaling Technology, Danvers, MA, USA; CN.4893); anti-SSEA-4, anti-tumour rejection antigen (TRA)-1-60 and anti-TRA-1-81 (1:100; Millipore Corp, Billerica, MA, USA; CN.SCR001); anti-CD31 (1:100; BD Pharmingen, San Diego, CA, USA; CN.558068), anti-βIII-Tubulin (1:100; Covance, Princeton, NJ; CN.MMS-435P), anti-α-fetoprotein (1:100; Sigma-Aldrich Co, St. Louis, MO, USA; CN.A8452), anti-AR (1:500; AR-N20: sc-816; Santa Cruz Biotech, Santa Cruz, CA, USA), anti-PSA (1:500, A67-B/E3: sc-7316; Santa Cruz Biotech, Santa Cruz, CA, USA) and anti-UPIb (1:100; Santa Cruz Biotech, Santa Cruz, CA, USA;, CN.sc-15174). Secondary antibodies used were Alexa546-conjugated goat anti-mouse, Alexa488-conjugated goat anti-mouse, and Alexa568-conjugated rabbit anti-goat (all at 1:400; Invitrogen Corp, Carlsbad, CA, USA). The nuclei were counterstained with 4’,6-diamidino-2-phenylindole (DAPI) and cells were mounted (Vectashield; Vector Laboratories Inc, Burlingame, CA, USA). Images were obtained using a confocal laser scanning microscopy system (Nikon Corp, Tokyo, Japan).

***In vitro differentiation (embryoid body formation)***

For embryoid body (EB) formation, iPSCs were transferred to low-adherence tissue culture plates (Thermo Scientific Sterilin; Thermo Fisher Scientific Inc, Waltham, MA, USA) in differentiation medium (80% Knockout-DMEM [Dulbecco’s Modified Eagle Medium], 20% foetal calf serum, 1% GlutaMAX-I Supplement, 1% MEM nonessential amino acids, 1% penicillin-streptomycin) and cultured on low-adhesion plates for 7–8 d. Next, the EBs were transferred onto 0.01% gelatin-coated plates for another 7–8 d before immunostaining.

***In vivo differentiation (teratoma formation)***

The human iPSCs were collected from MEF feeders by trypsinisation. The 1500 iPSCs were mixed with respective bladder mesenchyme or urogenital sinus mesenchyme isolated from 18-d-old embryonic Sprague-Dawley rat foetuses (Harlan Laboratories Inc, Indianapolis, IN, USA) as previously described [2,3]. Cells were mixed in type I collagen and, after overnight incubation at 37°C, were engrafted under the renal capsule of intact male CB17Icr/Hsd-severe combined immune deficient mice (Harlan Laboratories Inc, Indianapolis, IN, USA). Host mice were sacrificed after 12 wk. Tissues were fixed in formalin overnight, then were paraffin-embedded, sectioned, and stained with haematoxylin and eosin.

***Differentiation of human prostate induced pluripotent stem cells in vitro***

To induce prostate differentiation, prostate iPSCs (Pro-iPSCs) were suspended in prostate-specific differentiation medium (primary prostate stroma-conditioned medium was used and cells were fed with fresh conditioned medium every 48 h) for 3 wk. Three-dimensional EB structures that formed were transferred in the same medium for another 14 d to 0.01% gelatin-coated plates, with supplementation with 10 nM dihydrotestosterone. Subsequently, for antibody staining for Pro-iPSC differentiation, epithelial enrichment was undertaken using EpCAM immunomagnetic sorting (Miltenyi Biotec, Auburn, CA, USA).

*6.4. Differentiation of human urinary tract induced pluripotent stem cells in vitro*

To induce differentiation, both urinary tract iPSCs (UT-iPSCs) and Skin-iPSCs were removed from feeder cells and treated with conditioned medium for 14 d. Conditioned medium was collected from human urothelial cells or stroma cells derived from the primary culture of bladder or ureteric biopsies. First, confluent epithelial or stromal cultures were washed with phosphate-buffered saline and fresh medium was added (supplemented KSFM or RPMI medium as outlined in subsection 1.1). Every 24 h, the supernatant medium was collected, centrifuged at 500 g for 5 min, filtrated with a 0.2-µm syringe membrane filter (Millipore Corp, Billerica, MA, USA). The urothelial-conditioned medium was diluted to one-third volume with fresh DMEM, resulting in a final serum concentration of 2%, and the stroma-conditioned medium was diluted with an equal volume of fresh RPMI medium 10% FCS, as previously described in an optimised protocol [4].

**Supplement references**

[1] Takahashi K, Tanabe K, Ohnuki M, et al. Induction of pluripotent stem cells from adult human fibroblasts by defined factors. Cell 2007;131:861–72.

[2] Franco OE, Jiang M, Strand DW, et al. Altered TGF-beta signaling in a subpopulation of human stromal cells promotes prostatic carcinogenesis. Cancer Research 2011;71:1272–81.

[3] Oottamasathien S, Wang Y, Williams K, et al. Directed differentiation of embryonic stem cells into bladder tissue. Dev Biol 2007;304:556–66.

[4] Tian H, Bharadwaj S, Liu Y, Ma PX, Atala A, Zhang Y. Differentiation of human bone marrow mesenchymal stem cells into bladder cells: potential for urological tissue engineering. Tissue Eng Part A 2010;16:1769–79.
